# Western Ontario Veterinary Medical Association

**Published:** 1898-02

**Authors:** 


### SOCIETY PROCEEDINGS.

### WESTERN ONTARIO VETERINARY MEDICAL ASSOCIATION.

THIS Association met at the Masonic House, Stratford, on December 31, 1897. Meeting opened in the mornin g at the appointed hour. The President, Dr. Gibbs, occupied the chair and called the meeting to order. Minutes of the previous meeting were read and approved.

After the usual routine of business the report of the committee to attend the Ontario Veterinary Medical Association, held on December 24th, at Toronto, was taken up, and Dr. Black, of Clinton, who was one of the committee, was called on to report. He referred to the petition that was sent to the Minister, Hon. John Dryden, some time in March, 1897, in search of better legal protection. The minister at first seemed to be interested in the profession, and he gave us to understand that we were asking nothing but what we deserved as a profession, and now he slaps us in the face by appointing Lieut.-Col. McCrae as a public instructor in the use of tuberculin. Col. McCrae, it was claimed, was a layman, and did not know anything about the subject of tuberculosis or the tuberculin-test. He also stated that by the look of things at the present time, the government was not interested in our profession as to give us better legal protection, and that each and every one of us see the member for his district and try to get the members to bring up this bill at the next Parliament sitting.

Drs. Gibb, of St. Mary's, and Steele, of Stratford, also referred to this bill, and agreed with Dr. Black in his statement, and also referred to the fact that Lieut.-Col. McCrae was not qualified for this position as to the use of the tuberculin-test, as he was not a qualified veterinary surgeon, and that the tuberculin-test needs to be safeguarded by certain precautions not possible to those without professional experience.

Moved by Dr. McMaster, of Stratford, seconded by Dr. Greenwood, that we adjourn until one o'clock ; carried.

The afternoon session of the meeting opened at one o'clock sharp, and most of the time was taken up in reading papers.

Dr. C. Kirkton read a paper on "Parturient Apoplexy, its Causes, Symptoms, and Treatment." This paper was the subject of much discussion. Dr. Gibbs, of St. Mary's, also referred to parturient apoplexy, and stated that he had the opportunity of seeing it three times in the same animal, and was always successful in his treatment. Dr. Black, of Cifton, read a paper on "Septic Metritis in a Young Heifer," and related its causes, symptoms, treatment, etc. Dr. Gibbs, of St. Mary's, read a paper on "Swelled Legs" of the horse, such as dropsical and inflammatory swellings.

The active participants of the meeting were as follows: Drs. Hodings, Clark, McMaster, Steele, Caw, Manser, Greenwood, Gibbs, Burger, Eckert, and Wagner.

Moved by Dr. Burges that we adjourn ; seconded ; carried.

